# Musical experience strengthens the neural representation of sounds important for communication in middle-aged adults

**DOI:** 10.3389/fnagi.2012.00030

**Published:** 2012-11-23

**Authors:** Alexandra Parbery-Clark, Samira Anderson, Emily Hittner, Nina Kraus

**Affiliations:** ^1^Auditory Neuroscience Laboratory, Northwestern UniversityEvanston, IL, USA; ^2^Communication Sciences, Northwestern UniversityEvanston, IL, USA; ^3^Institute for Neuroscience, Northwestern UniversityEvanston, IL, USA; ^4^Neurobiology and Physiology, Institute for Neuroscience, Northwestern UniversityEvanston, IL, USA; ^5^Otolaryngology, Northwestern UniversityEvanston, IL, USA

**Keywords:** auditory, brainstem, musical experience, speech in noise, aging, musicians

## Abstract

Older adults frequently complain that while they can hear a person talking, they cannot understand what is being said; this difficulty is exacerbated by background noise. Peripheral hearing loss cannot fully account for this age-related decline in speech-in-noise ability, as declines in central processing also contribute to this problem. Given that musicians have enhanced speech-in-noise perception, we aimed to define the effects of musical experience on subcortical responses to speech and speech-in-noise perception in middle-aged adults. Results reveal that musicians have enhanced neural encoding of speech in quiet and noisy settings. Enhancements include faster neural response timing, higher neural response consistency, more robust encoding of speech harmonics, and greater neural precision. Taken together, we suggest that musical experience provides perceptual benefits in an aging population by strengthening the underlying neural pathways necessary for the accurate representation of important temporal and spectral features of sound.

## Introduction

Hearing speech in a noisy environment is difficult for everyone, yet older adults are particularly vulnerable to the effects of background noise (Gordon-Salant and Fitzgibbons, 1995). Given that everyday activities often occur in noisy environments, speech-in-noise perception is an important aspect of daily communication. Indeed, difficulty hearing in noise is one of the top complaints of older adults (Tremblay et al., 2003; Yueh et al., 2003). Additionally, their reduced ability to hear in noise may lead to the avoidance of social situations where noise is present, resulting in social isolation and decreased quality of life (Heine and Browning, 2002). With widespread population aging (Vincent and Velkoff, 2010), it is becoming increasingly pressing to understand the age-related changes in communication skills as well as the underlying biology that contributes to these communication problems.

Aging has a pervasive impact on the neural encoding of sound, with delayed neural responses and decreased neural precision (Walton et al., 1998; Burkard and Sims, 2001; Finlayson, 2002; Tremblay et al., 2003; Lister et al., 2011; Parthasarathy and Bartlett, 2011; Recanzone et al., 2011; Vander Werff and Burns, 2011; Wang et al., 2011; Anderson et al., 2012; Konrad-Martin et al., 2012; Parbery-Clark et al., 2012). While it was once thought that these effects were an obligatory trajectory of aging, an increasing body of work contradicts this notion (Thomas and Baker, 2012). Instead, studies using animal models have suggested that windows of critical period plasticity can be reopened for learning (Zhou et al., 2011) and that age-related declines are reversed with training (de Villers-Sidani et al., 2010). Recently, we demonstrated that lifelong musical training similarly prevents such declines (Parbery-Clark et al., 2012), suggesting that intensive auditory experience may act in some capacity as an “aging antidote.” The study of aging musicians may therefore inform what constitutes “optimal aging,” fostering the development of remediation strategies.

Intensive auditory experience, such as that offered by musical training, enhances brain systems underlying the neural encoding of communication sounds (Pantev et al., 2003; Fujioka et al., 2004; Schon et al., 2004; Shahin et al., 2005; Magne et al., 2006; Moreno and Besson, 2006; Marques et al., 2007; Musacchia et al., 2007; Lee et al., 2009; Tervaniemi et al., 2009; as reviewed in Kraus and Chandrasekaran, 2010; Besson et al., 2011; Bidelman et al., 2011a,b; Chobert et al., 2011; Marie et al., 2011; Shahin, 2011), including those aspects of neural encoding that are crucial for hearing in noise in young adults and children (Parbery-Clark et al., 2009a, 2011b; Bidelman and Krishnan, 2010; Strait et al., in press). Despite evidence for a speech-in-noise advantage in older adult musicians (Parbery-Clark et al., 2011a; Zendel and Alain, 2011), the mechanism through which musical experience impacts the neural encoding of speech in noise in an older population is poorly understood. Here, we aimed to delineate the effects of musical experience on the neural encoding of speech in noise by assessing speech-evoked auditory brainstem responses (ABRs) in quiet and noise in a middle-aged population of musicians and nonmusicians.

We focused our analyses on neural response timing, spectral encoding, and phase-locking to the stimulus, both in terms of the temporal envelope and higher-frequency components, because these elements decline with age (Anderson et al., 2012; Parbery-Clark et al., 2012; Ruggles et al., 2012), yet are enhanced in young musicians (Musacchia et al., 2007; Parbery-Clark et al., 2009a; Strait et al., 2012). Additionally these particular neural response components are important contributors to speech-in-noise perception in young adults and children. For example, there is a well-defined relationship between neural response timing and hearing in noise, with earlier response timing relating with improved speech-in-noise perception (Parbery-Clark et al., 2009a; Anderson et al., 2010). We also know that accurately perceiving and encoding the timbral structure unique to an individual's voice facilitates the creation of an auditory object (Griffiths and Warren, 2004; Shinn-Cunningham and Best, 2008) and its subsequent segregation from competing auditory streams (Iverson, 1995). Timbre perception is driven by both envelope and harmonic encoding (Krimphoff et al., 1994; McAdams et al., 1995) with both of these components known to play a role in hearing in noise (Swaminathan and Heinz, 2012; Strait et al., in press). As such, we hypothesized that musicians have enhanced neural encoding of the spectral and temporal components of the speech stimulus, resulting in a more precise neural representation of this signal. We were also interested in defining the relationship between these neural measures and indices of speech-in-noise perception in middle-aged listeners. To this aim we administered standardized (i.e., Hearing in Noise Test (HINT); Nilsson et al., 1994), and subjective (i.e., self-report questionnaire of perceived difficulties hearing in noise; Gatehouse and Noble, 2004) measures of hearing in noise, predicting that the neural measures would relate to speech-in-noise performance, providing at least a partial explanation for the middle-aged musician advantage for hearing in noise.

## Methods

### Participants

Forty-eight middle-aged adults (45–65 years, mean age 56 ± 5 years) participated. All subjects had normal hearing for octave frequencies from 0.125–4 kHz bilaterally ≤20 dB HL, pure-tone average ≤10 dB HL. Participants had no history of neurological or learning disorders, did not have asymmetric pure-tone thresholds (defined as ≥15 dB difference at two or more frequencies between ears) and demonstrated normal click-evoked ABRs (wave V latency ≤6.8 ms at 80 dB SPL). No participant reported a history of chemotherapy, taking ototoxic medications, major surgeries, or head trauma. In addition, all participants were native English speakers and had normal non-verbal IQ: Abbreviated Wechsler's Adult Scale of Intelligence's matrix reasoning subtest, (Wechsler, 1999). All experimental procedures were approved by the Northwestern University Institutional Review Board and participants provided informed consent.

Twenty-three subjects were categorized as musicians, having started musical training before the age of nine and consistently engaged in musical activities a minimum of three times a week throughout their lifetimes. For information relating to participants' music practice histories, see Table 1. Twenty-five subjects were categorized as nonmusicians with 17 having had no musical training and eight having fewer than 4 years of musical experience. The groups did not differ in age, hearing thresholds, sex, or non-verbal IQ (all *P* > 0.1; Table 2). Participants were also matched on measures of physical activity [*F*_(1, 47)_ = 0.032, *p* = 0.858], assessed by asking participants to describe the type and quantity of physical activity they engaged in each week. To account for varying types of physical activity, “walking” and “biking” were given half values while “running,” “weight training,” and other more vigorous activities were given full values. From these values, the total hours of physical activity per week was calculated for each participant. Participants were then assigned a value based on their overall activity level: 0 (<1 h/week), 1 (1–2 h/week), 2 (2–3 h/week), 3 (3–4 h/week), or 4 (>4 h/week); Table 2. Two musicians and three nonmusicians were left-handed. In terms of alcohol consumption, 4 musicians and 6 nonmusicians reported never drinking.

**Table 1:** **Participants' musical practice history**.

**Musicians**	**Years of musical**	**Age onset,**	**Instrument**
	**experience**	**years**	
1	56	5	Violin
2	49	6	Violin
3	43	8	Violin
4	38	9	Violin
5	48	9	Violin
6	54	6	Piano/Violin
7	46	4	Piano/Violin
8	46	6	Piano/French Horn
9	50	7	Piano/French Horn
10	52	6	Piano/Cello
11	51	9	Piano/Viola
12	57	6	Saxophone/Clarinet
13	50	6	Piano/Trombone
14	57	5	Piano
15	45	6	Piano
16	50	6	Piano
17	51	9	Piano
18	49	5	Piano
19	58	5	Piano
20	45	8	Piano
21	52	6	Violin
22	39	6	Piano
23	42	7	Piano
Mean	49	6.5	–

Age at which musical training began, years of musical training and major instrument(s) are indicated for all musician participants.

**Table 2:** **Participant characteristics: means (with SDs) for the musician and nonmusician groups are listed for age, pure-tone averages (0.5–4 kHz HL), click wave V latencies, non-verbal IQ percentiles (WASI Matrix Reasoning Subtest), and physical activity**.

	**Musicians (*N* = 23)**	**Nonmusicians (*N* = 25)**
Age (years)	55.2 (4.97)	57.3 (5.39)
PTA (dB HL)	8.97 (2.10)	9.60 (3.85)
Click (ms) – wave V	5.32 (1.70)	5.41 (1.32)
IQ (percentile)	81.00 (20.51)	81.89 (20.93)
Physical activity	2.13 (1.29)	2.20 (1.38)

### Electrophysiology

#### Stimulus

**Figure 1:**
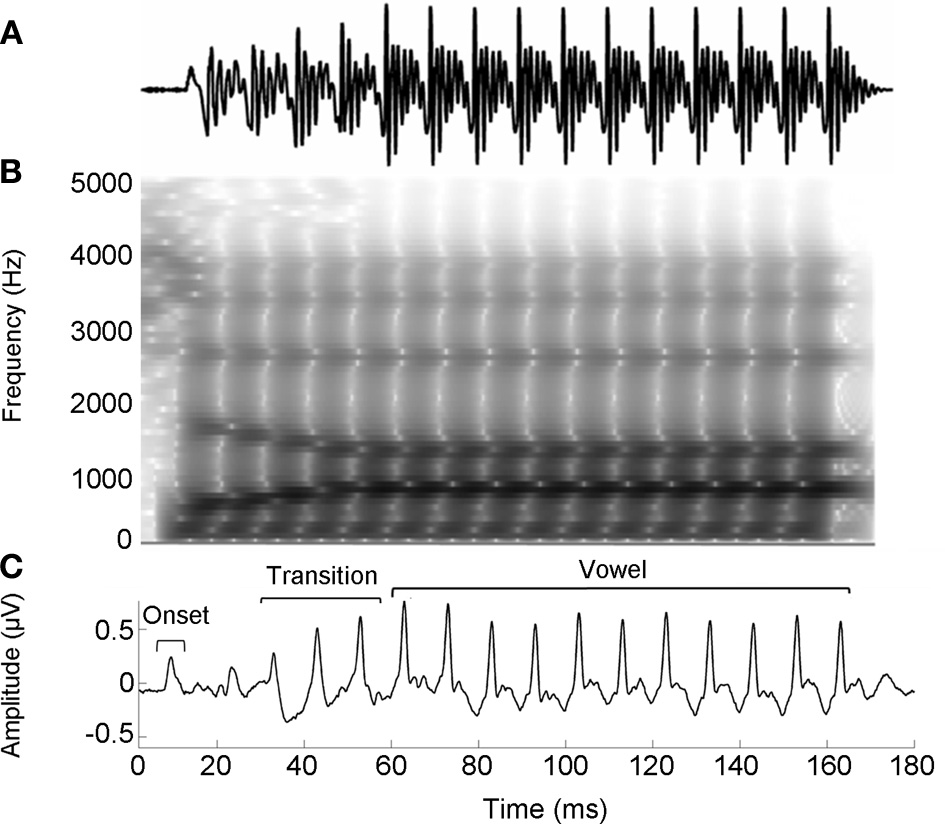
**Stimulus waveform (A), spectrogram (B), and group average response (C) for the speech syllable /da/.** The group average response plotted is the older musician response in quiet.

The speech stimulus was a 170 ms six-formant speech syllable /da/ synthesized at a 20 kHz sampling rate. This syllable has a steady fundamental frequency (*F*_0_ = 100 Hz) except for an initial 5 ms (onset) burst. During the first 50 ms (transition from the stop burst /d/ to the vowel /a/) the lower three formants change over time (*F*_1_, 400–720 Hz; *F*_2_, 1700–1240 Hz; *F*_3_, 2580–2500 Hz) but stabilize for the 120 ms steady-state vowel. The upper three formants are constant throughout (*F*_4_, 3300 Hz; *F*_5_, 3750 Hz; *F*_6_, 4900 Hz; See Figure 1). The /da/ was chosen because it combines a transient (the /d/) and periodic (the /a/) segment, two acoustic features which have been extensively studied using auditory brainstem responses (ABRs) (Skoe and Kraus, 2010). Additionally, stop consonants pose perceptual challenges to both young and older listeners (Miller and Nicely, 1955; Ohde and Abou-Khalil, 2001).

***Electrophysiologic recording parameters and procedure***. ABRs were differentially recorded at a 20 kHz sampling rate using Ag-AgCl electrodes in a vertical montage (Cz active, FPz ground and linked-earlobe references) in Neuroscan Acquire 4.3 (Compumedics, Inc., Charlotte, NC). Contact impedance was 2 kΩ or less across all electrodes. Stimuli were presented binaurally in alternating polarities at 80 dB SPL with an 83 ms inter-stimulus interval (Scan 2, Compumedics, Inc.) through ER-3 insert earphones (Etymotic Research, Inc., Elk Grove Village, IL). During the recording session (26 ± 2 min) subjects watched a silent, captioned movie of their choice to facilitate a restful state.

***Data reduction***. Responses were band-pass filtered offline from 70 to 2000 Hz in MATLAB (12 dB/octave, zero phase-shift; The Mathworks, Inc., Natick, MA) and epoched using a −40 to 213 ms time window referenced to stimulus onset. Any sweep with an amplitude beyond ±35 μV was considered artifact and rejected, resulting in a total of 6000 response trials for each subject. The responses of the two polarities were added to minimize the influence of cochlear microphonic and stimulus artifact on the response (Aiken and Picton, 2008). Response amplitudes were baseline corrected to the prestimulus period.

#### Timing

**Figure 2:**
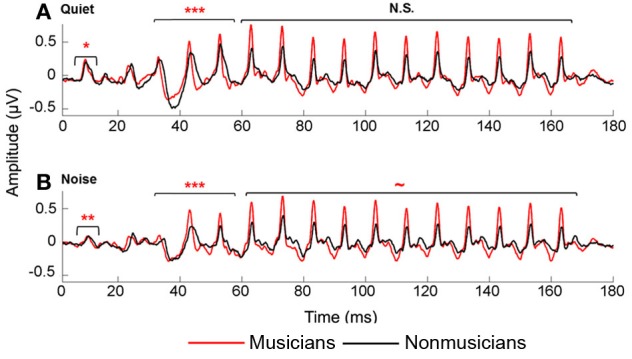
**Average brainstem responses to /da/ in musician (red) and nonmusician (black) middle-aged adults in quiet (A) and noise (B).** In quiet, musicians had earlier neural response timing for the onset and transition portion; in noise, musicians had earlier neural responses for the onset and transition, with a marginally significant trend for the vowel. ~*p* < 0.1, ^*^p < 0.05, ^**^p < 0.01, ^***^p < 0.001.

We manually identified peaks in the subcortical responses generated by synchronous neural firing to the speech syllable /da/. The identification provides each peak's latency and amplitude. Peaks were labeled according to stimulus onset at time 0 ms such that a peak occurring at ~33–34 ms after onset would be called Peak 33. The first major peak, in response to the onset of the sound, was identified as Peak 9, those that correspond to the transition were peaks 33, 43, and 53, and to the vowel were peaks 63–163 at 10 ms intervals (Figure 2). Two individuals who were blind to participant group independently identified each peak. An additional peak-picker confirmed peak identification and resolved disagreement between the two.

All participants had distinct transition and vowel peaks for both the quiet and noise conditions, but onsets were absent for one participant (a nonmusician) in the quiet condition and for four participants (1 musician, 3 nonmusicians) in noise. Statistical analyses for onset peak latencies only included participants with discernible peaks in both quiet and noise (*n* = 44). For correlational analyses between peak timing and speech-in-noise perception, composite peak timing scores were created for the transition and the vowel regions. These composite scores were calculated by taking the average latency of peaks 33–53 for the transition and 63–163 for the vowel which when reported are denoted as transition_mean_ and vowel_mean_.

#### Spectral representation: fundamental frequency and harmonics

The neural encoding of the stimulus spectrum was calculated using a fast Fourier transform in MATLAB. The average spectral amplitudes relating to the transition (20–60 ms) and the vowel (60–170 ms) regions were determined by using 20-Hz bins centered around the frequencies of interest which included the fundamental frequency (*F*_0_) and its subsequent integer harmonics H_2_–H_10_ (200–1000 Hz, whole integer multiples of the *F*_0_). These values were used for all statistical analyses except for correlations for which we created a composite harmonic score by averaging the H_2_–H_10_ bins, representing the strength of overall harmonic encoding.

#### Stimulus-to-response—envelope analyses and waveform correlation

To measure the effect of noise on the neural response we employed two types of stimulus-to-response correlations. The first was to assess the effect of noise on the global envelope encoding by calculating the degree of correlation between the envelope of the stimulus and each participant's neural envelope encoding in the quiet and noise conditions. The second was to assess the effect of noise on neural response morphology by calculating the degree of similarity between the stimulus waveform and each participant's neural response in both the quiet and noise conditions. For this second analysis, two time ranges were chosen corresponding to the transition and the vowel. In both cases, we band-pass filtered the stimulus to match the brainstem response characteristics (70–2000 Hz). For the envelope analyses we obtained the broadband amplitude envelopes by performing a Hilbert transform on the stimulus and response waveforms and low-pass filtering at 200 Hz. To calculate the correlations between the stimulus and the responses we used the xcorr function in MATLAB (Skoe and Kraus, 2010). In both cases, the degree of similarity was calculated by shifting the stimulus waveform over 7–12 ms range relative to the regions of interest until a maximum correlation value was found. The 7–12 ms time lag was chosen because it encompasses the stimulus transmission delay (from the ER-3 transducer and ear insert ~1.1 ms) and the neural lag between the cochlea and the rostral brainstem. Average *r*-values were Fisher transformed for statistical analysis. Higher *r*-values indicate greater degrees of correlation.

***Response consistency***. Response consistency was calculated across trials over the length of the recording period (i.e., 6000 sweeps) by creating a composite response consistency score for each subject. Specifically, we created 300 randomly selected pairs of 3000, non-overlapping sweep sub-averages. To determine the degree of similarity between the individual pair sets, each pair of sub-averages was cross-correlated in MATLAB to generate a Pearson's correlation coefficient. This process was performed for each of the 300 pairs and the final value represents the average of the 300 correlation values. Response consistency was computed for the two time regions of interest: the transition and the vowel. Average *r*-values were Fisher transformed for statistical analysis. Higher *r*-values indicate greater degrees of correlation.

***Hearing in noise ability***. We used the Hearing in Noise Test (*HINT*; Bio-logic Systems Corp; Mundelein, IL) (Nilsson et al., 1994) to assess speech perception in noise. HINT is an adaptive test of speech recognition that measures speech perception ability in noise. During this test participants repeated short and semantically and syntactically simple sentences (e.g., *she stood near the window*) that were presented in speech-shaped background noise. Speech stimuli consisted of Bamford-Kowal-Bench (Bench et al., 1979) sentences (12 lists of 20 sentences) spoken by a male and were presented in free field. Participants sat one meter from the loudspeaker from which the target sentences and the noise originated at a 0° azimuth. The noise presentation level was fixed at 65 dB SPL and the program adjusted perceptual difficulty by increasing or decreasing the intensity level of the target sentences until the threshold signal-to-noise ratio (SNR) was determined. Perceptual speech-in-noise thresholds were defined as the level difference (in dB) between the speech and the noise presentation levels at which 50% of sentences are correctly repeated. A lower SNR indicates better performance.

***Self-reported hearing in noise ability***. We administered the Speech subscale of the Speech, Spatial, and Qualities Questionnaire (Gatehouse and Noble, 2004) to gauge an individual's perception of their hearing in noise. This questionnaire consists of 14 questions about hearing performance in various environments using a 10-point Likert scale. See Table 3 for a complete list of the questions.

**Table 3:** **Means, standard deviations, and significance values for the musician and nonmusicians groups' self-assessment of their speech perception and speech-in-noise abilities**.

**Questions**	**Musicians**	**Nonmusicians**	***p*-value**
You are talking with one other person and there is a TV on in the same room. Without turning the TV down, can you follow what the person you're talking to says?	9.03 (1.4)	7.92 (1.91)	0.010
You are talking with one other person in a quiet, carpeted lounge-room. Can you follow what the other person says?	9.75 (0.39)	9.56 (0.92)	0.970
You are in a group of about five people, sitting round a table. It is an otherwise quiet place. You can see everyone else in the group. Can you follow the conversation?	9.70 (0.67)	9.32 (0.75)	0.047
You are in a group of about five people in a busy restaurant. You can see everyone else in the group. Can you follow the conversation?	8.81 (1.12)	8.04 (1.59)	0.099
You are talking with one other person. There is continuous background noise, such as a fan or running water. Can you follow what the person says?	9.48 (0.82)	8.52 (1.62)	0.011
You are in a group of about five people in a busy restaurant. You cannot see everyone else in the group. Can you follow the conversation?	8.28 (1.6)	7.04 (2.09)	0.029
You are talking to someone in a place where there are a lot of echoes, such as a church or railway terminus building. Can you follow what the other person says?	8.90 (1.49)	7.92 (1.15)	0.005
Can you have a conversation with someone when another person is speaking whose voice is the same pitch as the person you're talking to?	8.94 (1.00)	7.88 (1.71)	0.029
Can you have a conversation with someone when another person is speaking whose voice is different in pitch from the person you're talking to?	9.11 (1.01)	6.6 (1.76)	0.017
You are listening to someone talking to you, while at the same time trying to follow the news on TV. Can you follow what both people are saying?	7.71 (2.26)	7.4 (2.00)	0.033
You are in conversation with one person in a room where there are many other people talking. Can you follow what the person you are talking to is saying?	8.35 (1.72)	8.2 (1.67)	0.041
You are with a group and the conversation switches from one person to another. Can you easily follow the conversation without missing the start of what each new speaker is saying?	8.97 (1.55)	9.32 (1.66)	0.059
Can you easily have a conversation on the telephone?	9.71 (0.59)	9.32 (0.80)	0.051
You are listening to someone on the telephone and someone next to you starts talking. Can you follow what's being said by both speakers?	7.36 (2.08)	6.76 (1.33)	0.133

These questions are part of the Speech, Spatial, and Qualities Assessment questionnaire. Note that not all of the above questions relate to hearing in noise.

### Statistical analyses

All statistical analyses were conducted in SPSS Version 18.0 (SPSS Inc., Chicago, IL). Repeated measure analyses of variance (RMANOVA) were used for group (musician vs. nonmusician) × condition (quiet vs. noise) comparisons for latency, spectral representation, stimulus-to-response correlations, envelope encoding, and response consistency. Univariate analyses of variance were used for behavioral measures. *Post-hoc* tests were used when appropriate. To assess relationships among variables, Pearson *r* correlations were used. Levene's test was used to ensure homogeneity of variance for all measures and the Shapiro-Wilk test was used to ensure that all variables were normally distributed. Bonferroni corrections for multiple comparisons were applied as appropriate; *p*-values reflect two-tailed tests. The SSQ (self-reported hearing in noise ability) was the only test that violated the assumption of normality. Neither log nor reciprocal transforms rendered these data normal. As such, we only used these data to quantify group differences using the non-parametric Mann-Whitney test; correlations with other variables were not explored.

## Results

### Summary of results

Musicians demonstrated greater speech-in-noise perception [HINT: *F*_(1, 47)_ = 20.276, *p* < 0.005; musicians mean: −3.16, SD 0.61; nonmusicians mean: −2.34, SD 0.63] and rated themselves as having less difficulty hearing in noise than nonmusicians as assessed by the SSQ (Table 3). Musicians exhibited more robust neural encoding of speech in both quiet and noise. Musicians had earlier neural response timing, greater neural representation of the stimulus harmonics as well as more precise phase-locking to the stimulus both in terms of temporal envelope and stimulus-to-response correlations. Musicians also demonstrated less neural response degradation in noise evidenced by smaller neural timing shifts and smaller decreases in neural response consistency. We also found that specific neural measures such as earlier neural response timing and more robust brainstem responses to speech correlated with better speech-in-noise performance as measured by HINT.

### Timing

Musicians demonstrated enhanced onset and transition timing in quiet and limited degradative effects of background noise for all aspects of neural timing. To quantify effects of musicianship and noise on neural response timing, we divided the neural response into three time regions: onset, transition, and vowel. We performed a mixed-model repeated-measures ANOVA (RMANOVA) 2 group (musician/nonmusician) × 2 condition (quiet/noise) with latencies in the three distinct time regions entered as dependent variables. Noise delayed peak timing across all time regions [onset: *F*_(1, 42)_ = 98.008, *p* < 0.001; transition: *F*_(1, 46)_ = 19.113, *p* < 0.001; vowel *F*_(1, 46)_ = 2.375, *p* = 0.025]. Musicians demonstrated earlier neural response timing for both the onset [*F*_(1, 42)_ = 11.080, *p* = 0.002] and the transition [*F*_(1, 46)_ = 13.219, *p* < 0.001] but not for the vowel [*F*_(1, 46)_ = 1.471, *p* = 0.185]. A significant group-by-condition interaction was found for all three time regions [onset: *F*_(1, 42)_ = 4.822, *p* = 0.034; transition: *F*_(1, 46)_ = 3.668, *p* < 0.019; vowel *F*_(1, 46)_ = 2.053, *p* = 0.050]. *Post-hoc* tests revealed that musicians had significantly earlier responses in both quiet and noise conditions for the onset and transition [Onset_quiet_: *F*_(1, 42)_ = 4.521, *p* = 0.039; Onset_noise_: *F*_(1, 42)_ = 12.720, *p* = 0.001; Transition_quiet_: *F*_(1, 46)_ = 10.459, *p* < 0.001; Transition_noise_: *F*_(1, 46)_ = 11.786, *p* < 0.001], whereas for the steady-state musicians and nonmusicians were equated in quiet but musicians were earlier in noise [Vowel_quiet_: *F*_(1, 46)_ = 1.423, *p* = 0.205; Vowel_noise_: *F*_(1, 46)_ = 1.912, *p* = 0.071]. In summary, musicians demonstrate earlier response timing in quiet for the onset and the transition but not the vowel. We also find that the addition of background noise delays neural responses for both groups, but that musicians' responses shifted less than those of nonmusicians (Figure 2).

### Spectral representation

#### Harmonics

**Figure 3:**
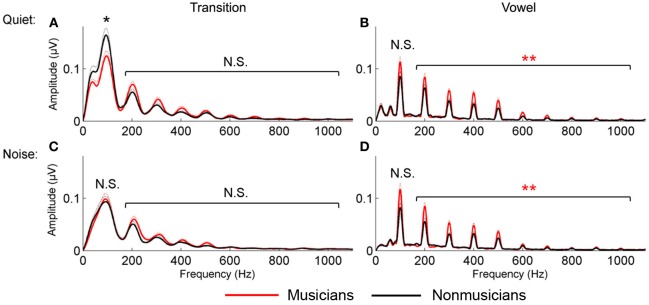
**Spectral encoding for the transition (A and C) and vowel (B and D) in quiet (A and B) and noise (C and D).** Musicians (red) demonstrated enhanced spectral encoding for the vowel in both quiet and noise; nonmusicians (black) had greater *F*_0_ encoding in the transition in quiet only. ^*^p < 0.05, ^**^p < 0.01.

For the vowel, in both quiet and noise, musicians demonstrated more robust auditory brainstem representation of the harmonics than nonmusicians; no musician advantage was found for the neural encoding of the harmonics in the transition. A 2 (musician/nonmusician) × 2 condition (quiet/noise) × 9 harmonics_H2−H10_ RMANOVA revealed a main effect of noise and musicianship on responses to the vowel, with noise reducing spectral amplitudes, [*F*_(1, 46)_ = 4.655, *p* < 0.001] and musicians having greater spectral amplitudes than the nonmusicians [*F*_(1, 46)_ = 2.831, *p* = 0.012] but no noise × musicianship interaction [*F*_(1, 46)_ = 1.476, *p* = 0.192]. For the transition, again noise resulted in a reduction in harmonic amplitude [*F*_(1, 46)_ = 7.418, *p* < 0.001] but there was no musician advantage [*F*_(1, 46)_ = 1.046, *p* = 0.423] nor a significant noise × musicianship interaction [*F*_(1, 46)_ = 1.001, *p* = 0.456; Figure 3].

#### Fundamental frequency (F_0_)

For the vowel, in both quiet and noise, musicians demonstrated a trend toward a greater representation of the fundamental frequency. This was not observed for responses to the transition. A 2 group (musician/nonmusician) × 2 condition (quiet/noise) RMANOVA revealed a weak trend for musicianship [*F*_(1, 46)_ = 2.900, *p* = 0.095] but no main effect of noise [*F*_(1, 46)_ = 0.089, *p* = 0.767] nor noise × musicianship interaction [*F*_(1, 46)_ = 1.404, *p* = 0.242]. For the transition, there was a main effect of noise [*F*_(1, 46)_ = 48.977, *p* < 0.001], no main effect of musicianship [*F*_(1, 46)_ = 0.004, *p* = 0.300] but a significant interaction [*F*_(1, 46)_ = 7.063, *p* = 0.011]. *Post-hoc* tests revealed that nonmusicians had greater representation of the F_0_ in quiet [*F*_(1, 46)_ = 4.103, *p* = 0.049] but not in noise [*F*_(1, 46)_ = 0.070, *p* = 0.792; Figure 3].

### Stimulus to response

#### Envelope analyses

**Figure 4:**
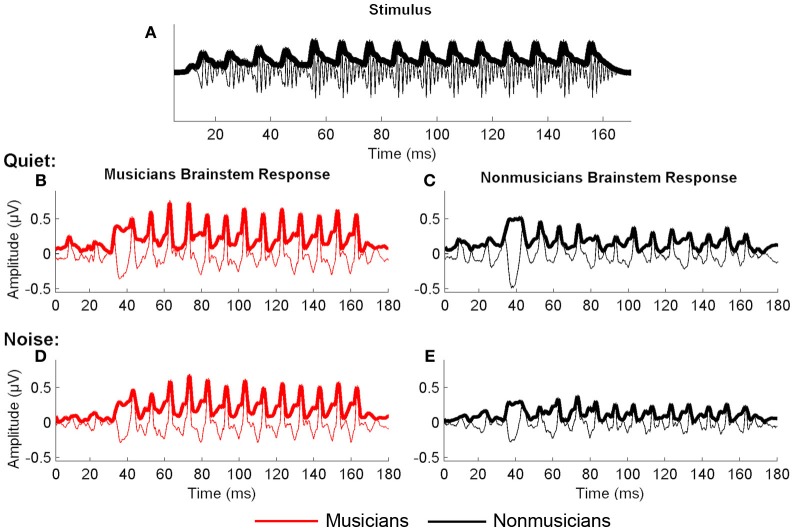
**Envelope correlations between the stimulus (A) and the responses from the two conditions: quiet (B and C) and noise (D and E).** The neural encoding of the stimulus envelope was greater in musicians (red) than nonmusicians (black) for both quiet and noise.

In both quiet and noise, musicians had better neural representation of the stimulus envelope [Figure 4; *F*_(1, 46)_ = 23.893, *p* < 0.001; Table 4]. Noise had a significant effect on envelope encoding, in that for both groups, envelope encoding got stronger in noise [*F*_(1, 46)_ = 4.665, *p* = 0.036; Table 5]. No significant noise × musicianship interaction was found [*F*_(1, 46)_ = 0.071, *p* = 0.792].

**Table 4:** **Stimulus-to-response (envelope and waveform) correlation values (Pearson *r*): means (with SDs) for the musicians and nonmusicians across the relevant time regions**.

**Time-range**	**Musicians**	**Nonmusicians**	***p*-value**
	**Stimulus-to-response**		
	**envelope correlations**		
Quiet			
Entire (5–180 ms)	0.66 (0.14)	0.42 (0.20)	<0.001
Noise			
Entire (5–180 ms)	0.69 (0.14)	0.47 (0.15)	<0.001
	**Stimulus-to-response**		
	**waveform correlations**		
Quiet			
Transition (20–60 ms)	0.244 (0.08)	0.21 (0.078)	0.167
Vowel (60–170 ms)	0.33 (0.39)	0.24 (0.083)	<0.001
Noise			
Transition (20–60 ms)	0.24 (0.08)	0.22 (0.08)	0.401
Vowel (60–170 ms)	0.32 (0.04)	0.23 (0.11)	<0.001

**Table 5:** **Response consistency scores (Pearson *r*-values): means (with SDs) for the musicians and nonmusicians across the transition and the vowel**.

**Time Range**	**Response consistency**		***p*-value**
	**Musicians**	**Nonmusicians**	
Quiet			
Transition (20–60 ms)	0.84 (0.08)	0.82 (0.14)	0.856
Vowel (60–170 ms)	0.86 (0.07)	0.73 (0.15)	0.001
Noise			
Transition (20–60 ms)	0.74 (0.18)	0.66 (0.19)	0.083
Vowel (60–170 ms)	0.83 (0.11)	0.68 (0.17)	0.001

Consistency measures are derived by correlating 300 randomly-selected pairs of 3000 sweeps from an individual's response.

#### Waveform correlation

Musicians demonstrated more precise neural representation of the vowel in both quiet and noise [*F*_(1, 46)_ = 20.290, *p* < 0.001; Table 4]. The addition of background noise degraded neural response morphology [*F*_(1, 46)_ = 5.492, *p* = 0.023], but no significant interaction was present [*F*_(1, 46)_ = 0.504, *p* = 0.481]. For the transition, no effect of noise, [*F*_(1, 46)_ = 5.492, *p* = 0.429], musicianship, [*F*_(1, 46)_ = 1.584, *p* = 0.215], or a significant interaction [*F*_(1, 46)_ = 0.504, *p* = 0.522] was found, suggesting that this particular analytical measure did not capture the degradation caused by noise in this time region.

#### Response consistency

Musicians had greater neural response consistency in both quiet and noise for the vowel [*F*_(1, 46)_ = 13.488, *p* = 0.001], despite the addition of noise resulting in a decline in response consistency for both groups [*F*_(1, 46)_ = 5.795, *p* < 0.020]. No significant noise × group interaction was present [*F*_(1, 46)_ = 0.022, *p* = 0.882]. For the transition, noise reduced response consistency [*F*_(1, 46)_ = 67.884, *p* < 0.001]; yet musicians did not demonstrate an overall enhancement in both quiet and noise conditions [*F*_(1, 46)_ = 0.803, *p* = 0.375]. Rather, there was a trending interaction [*F*_(1, 46)_ = 3.072, *p* = 0.086] with musicians and nonmusicians having equivalent response consistency in quiet [*F*_(1, 46)_ = 0.033, *p* = 0.856] but musicians having marginally greater response consistency in noise [*F*_(1, 46)_ = 3.133, *p* = 0.083; Table 5].

#### Brainstem-hearing in noise relationships

**Figure 5:**
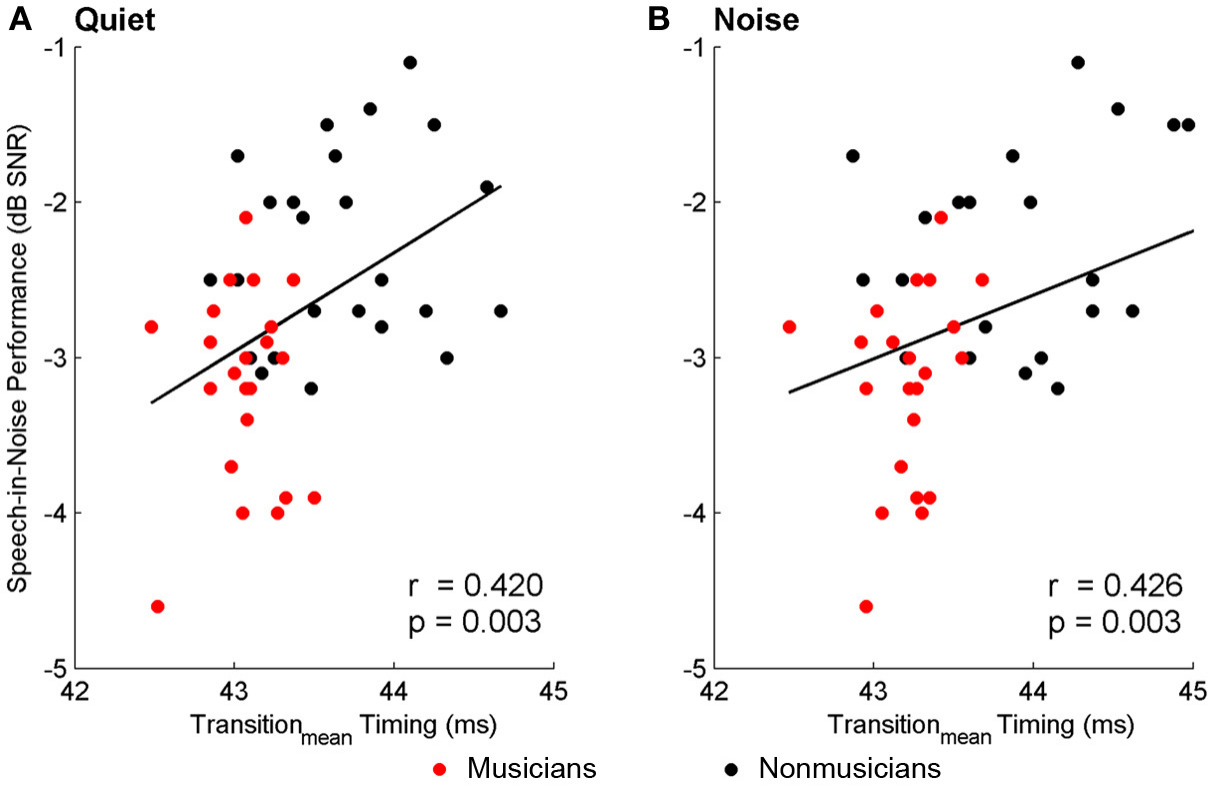
**Relationships between speech-in-noise performance and brainstem response timing.** Earlier neural response timing in the transition for both the quiet **(A)** and noise **(B)** conditions is associated with better hearing in noise. Similar relationships (not plotted here) were found for the neural response timing to the onset and the vowel; see text for more details. A lower, more negative speech-in-noise score is indicative of better performance.

**Figure 6:**
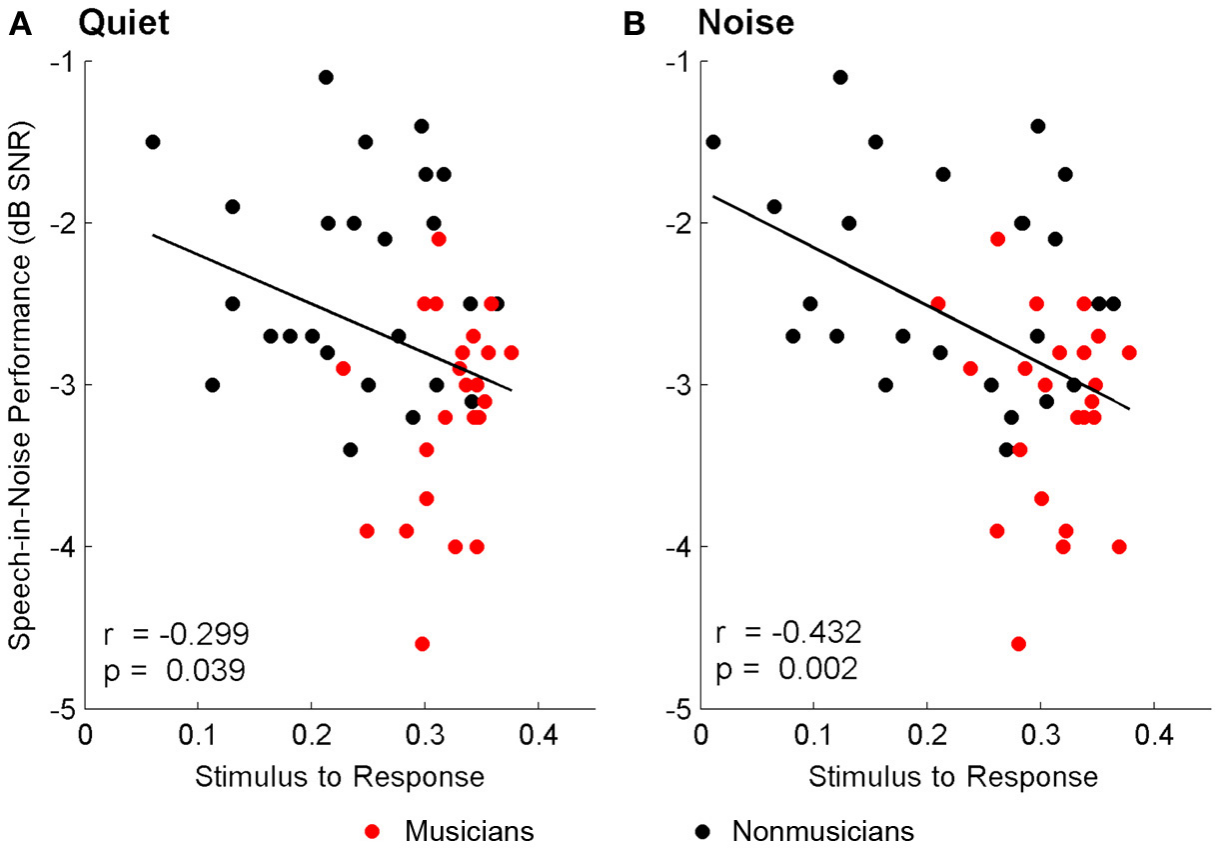
**Relationships between speech-in-noise performance and stimulus-to-response waveform (i.e., vowel) correlations.** Better hearing in noise was associated with higher stimulus-to-response correlations in quiet **(A)** and noise **(B)**, suggesting that greater precision in the brainstem's ability to represent the stimulus in both conditions is important for understanding speech in noise. A lower, more negative speech-in-noise score is indicative of better performance.

Brainstem measures in both quiet and noise related to speech-in-noise perception as measured by HINT. The accuracy with which the ABR represented the envelope of the speech sound related to HINT (envelope_quiet_: *r* = −0.278, *p* = 0.05; envelope_noise_: *r* = −0.346, *p* = 0.016). In all cases, earlier neural response latencies (Figure 5, Table 6) and greater SR_vowel_ correlations (Figure 6) were associated with better HINT scores. SR_transition_ correlations in quiet and noise were not related to speech-in-noise perception (all *p* > 0.1).

**Table 6:** **Correlations (with significance levels) between peak latency for the onset, transition, and vowel peaks for the two conditions (i.e., Quiet and Noise) and HINT**.

	**Quiet**			**Noise**		
	**Onset**	**Transition_mean_**	**Vowel_mean_**	**Onset**	**Transition_mean_**	**Vowel_mean_**
HINT	0.356 (0.014)	0.420 (0.003)	0.378 (0.008)	0.315 (0.038)	0.426 (0.003)	0.335 (0.020)

In all cases earlier response timing related to better speech-in-noise perception.

Response consistency also related to HINT. In quiet, the RC_vowel_ related with speech-in-noise perception (*r* = −0.307, *p* = 0.034) but not the RC_transition_ (*r* = −0.202, *p* = 0.169). In noise, the RC_transition_ related with hearing in noise (*r* = −0.291, *p* = 0.045) but not the vowel (*r* = −0.185, *p* = 0.208). Lastly, neither the representation of the F_0_ nor the harmonics directly related to speech-in-noise performance (all *p* > 0.1).

## Discussion

Here we show that middle-aged musicians have greater neural fidelity of the stimulus with faster neural response timing, better envelope encoding, greater neural representation of the stimulus harmonics as well as less neural degradation with the addition of background noise. These subcortical measures are all associated with better speech perception in noise. Furthermore, we reveal that middle-aged musicians rate their speech-in-noises abilities higher than nonmusicians, suggesting that musicians' communication skills are higher than nonmusicians in real-world listening environments. Taken together, these results indicate that musical experience in an older adult population is associated with more precise neural responses and greater resistance to the deleterious effects of background noise.

### More precise neural encoding relates with speech-in-noise perception

Hearing in noise relies on the ability to distinguish and track the target voice from the background noise, and recognizing the distinct timbral signature of a person is a key way to achieve this. Envelope and harmonic cues contribute to timbre (Krimphoff et al., 1994; McAdams et al., 1995), making them an important component of the neural code.

Envelope encoding, like stimulus-to-response correlations, is thought to represent the neural encoding of mid-to-high frequency neurons (Dau, 2003; Parbery-Clark et al., 2009a; Ruggles et al., 2012), thus providing a direct link between envelope encoding, the neural representation of higher harmonics and timbre. Furthermore, robust envelope and stimulus-to-response correlations are behaviorally relevant in that they facilitate listening in complex environments such as in background noise (Parbery-Clark et al., 2009a; Swaminathan and Heinz, 2012) or reverberant environments (Ruggles et al., 2012). Our results indicate that middle-aged musicians have stronger representation of envelope, stimulus-to-response and harmonic encoding than nonmusicians and we believe that the strengthened encoding of these spectral features may afford musicians the ability to better discern and segregate voices, giving them an advantage for speech-in-noise perception (Parbery-Clark et al., 2009a,b, 2011a; Zendel and Alain, 2011; Strait et al., in press). Throughout their training and subsequent musical experience, musicians spend countless hours attending to spectrally rich musical sounds, learning to use subtle differences in acoustic cues to discriminate instruments. Spectral information is of great behavioral relevance for musicians, with young adult musicians detecting slight harmonic differences as well as having a greater neural representation of harmonics (Koelsch et al., 1999; Shahin et al., 2005; Musacchia et al., 2008; Lee et al., 2009; Parbery-Clark et al., 2009a; Zendel and Alain, 2009). Our results indicate that older musicians also have a greater neural representation of the harmonics than nonmusicians suggesting that musical experience maintains spectral encoding despite the general trajectory of decline in the ability of the nervous system to represent spectral cues across the lifespan (Clinard et al., 2010; Ruggles et al., 2011, 2012; Anderson et al., 2012).

Middle-aged musicians demonstrate enhanced neural timing of speech in both quiet and noise—as has been found in child musicians (Strait et al., in press), whereas young adult musicians (Parbery-Clark et al., 2009a) only exhibit these enhancements in the more challenging of the two conditions—in noise. In explaining the developmental trajectory between child musicians to young adults, we propose that musical training during childhood accelerates the developmental trajectory of neural mechanisms underpinning the neural encoding of sound, as demonstrated by earlier neural response timing in child musicians, whether it be in the presence or absence of background noise (Strait et al., in press). By young adulthood, we suggest that nonmusicians have “*caught up*” with the musicians in that both groups are equated for response timing in quiet, even though musicians are still earlier in noise (Parbery-Clark et al., 2009a; Strait et al., in press). Here we extend this proposal to suggest that on the other side of the life cycle—that of aging—musical experience prevents declines in neural mechanisms that underlie neural encoding irrespective of the listening environment.

Our results highlight faster response timing in middle-aged musicians for the onset and transition—two parts of the response that decline with both age (Anderson et al., 2012; Parbery-Clark et al., 2012) and the introduction of noise (Cunningham et al., 2002; Parbery-Clark et al., 2009a; Anderson et al., 2010), are the most challenging in terms of perception (Miller and Nicely, 1955) and neural encoding (Anderson et al., 2010). Importantly, in quiet, there were no group differences for the vowel, indicating that the middle-aged nonmusician's neural responses are not globally delayed for response timing; rather, these effects were exclusively found in the response to the most complex portions of the sound (Parbery-Clark et al., 2012). The addition of background noise did result in a general delay for both groups; still, musicians' responses were delayed to a lesser extent. Musicians' decreased neural response degradation in noise was further evidenced by more consistent neural responses. Taken together, our results provide evidence for musical training across the life span having a pervasive effect on sensory and neural processing, maintaining neural function both in quiet and noisy conditions.

### Musicians: model of aging

To date, the majority of research supporting the use of musicians as a model of plasticity has focused on child or young adult populations (for review see: Münte et al., 2002; Zatorre and McGill, 2005; Habib and Besson, 2009; Kraus and Chandrasekaran, 2010). While this work has increased our understanding of the effects of music on the nervous system, the role of musical training in the older normal hearing adult population remains largely unexplored. Given that musical training strengthens those skills that decline with age, we argue that the musician's brain provides an optimal model for studying the effects of age on the nervous system. Aging declines are thought to start as early as middle age (Salthouse et al., 1996; Helfer and Vargo, 2009; Ruggles et al., 2011, 2012; Parbery-Clark et al., 2012) and are accompanied by a decrease in central nervous system function, which holds important implications for perceptual and cognitive skills (Craik and Salthouse, 2000). Given that aging musicians maintain an advantage over nonmusicians in terms of neural processing (Parbery-Clark et al., 2012), auditory perception (Parbery-Clark et al., 2011a; Zendel and Alain, 2011) and cognitive abilities (Hanna-Pladdy and MacKay, 2011; Parbery-Clark et al., 2011a; Hanna-Pladdy and Gajewski, 2012), older musicians may provide a means to better understand what contributes to successful aging.

The application of musical experience to the study of aging requires knowledge of the effects of aging on the nervous system. One of the major neurophysiological hallmarks of aging is delayed neural timing and decreased temporal processing (Walton et al., 1998; Burkard and Sims, 2001; Frisina, 2001; Finlayson, 2002; Tremblay et al., 2003; Frisina and Walton, 2006; Lister et al., 2011; Parthasarathy and Bartlett, 2011; Recanzone et al., 2011; Vander Werff and Burns, 2011; Wang et al., 2011; Anderson et al., 2012; Konrad-Martin et al., 2012; Parbery-Clark et al., 2012). These age-related deficits are caused, at least in part, by a decrease in inhibitory mechanisms. With aging, the inhibitory neurotransmitters that facilitate the accurate neural encoding of temporally dynamic and complex sounds (Walton et al., 1998; Caspary et al., 2002, 2008) as well as response consistency (Pichora-Fuller and Schneider, 1992) are reduced throughout the auditory pathway (Caspary et al., 1995, 2005; Wang et al., 2009; de Villers-Sidani et al., 2010; Hughes et al., 2010; Juarez-Salinas et al., 2010). Because the ABR requires a high-degree of neural synchronicity (Kraus et al., 2000), decreased neural consistency such as that caused by temporal jitter (Pichora-Fuller et al., 2007) or neural response variability (Turner et al., 2005; Yang et al., 2009) associated with aging can also contribute to delayed neural response timing and reduced spectral encoding (Anderson et al., 2012). Here we present musician advantages for neural response timing, spectral encoding, and neural response consistency—all factors known to decline with age. For these reasons, we propose that the study of the older musician may be beneficial in elucidating the specific neural components that are enhanced relative to nonmusicians or impervious to age-related declines—highlighting which aspects may be amenable to rehabilititation.

### Future directions

We document enhanced neural encoding in a normal hearing, middle-aged adult musician population. Because aging also results in a higher prevalence of hearing loss, it will be important to define how musical experience interacts in an older adult population with sensory hearing loss. Additionally, our earlier work demonstrated that young adult musicians (19–30 years) had minimal neural differences in quiet (Parbery-Clark et al., 2009a), yet the present results show striking group differences in a middle-aged group (45–65 years) for the same condition. Determining the time course of the neural changes that occur between these two age groups (i.e., young and middle-aged adults) will further our understanding of the effects of aging on the nervous system, as well as the role musicianship plays to offset these declines.

## Conclusions

We reveal strengthened neural encoding of the important acoustic ingredients for speech perception in noise for middle-aged musicians, potentially providing a neural basis for their behavioral advantage for hearing in noise.

### Conflict of interest statement

The authors declare that the research was conducted in the absence of any commercial or financial relationships that could be construed as a potential conflict of interest.
